# Leiomyoma originating from axilla: A rare case report and differential diagnosis

**DOI:** 10.1097/MD.0000000000004402

**Published:** 2016-07-29

**Authors:** Ho Jun Kim, Sang Oon Baek, Eun Young Rha, Jun Yong Lee, Hyun Ho Han

**Affiliations:** Department of Plastic and Reconstructive Surgery, Incheon St. Mary's Hospital, college of Medicine, The Catholic University of Korea, Seoul, Republic of Korea.

**Keywords:** axilla, deep soft tissue, differential diagnosis, leiomyoma

## Abstract

**Introduction:**

Leiomyoma is a form of benign tumor originated in hypertrophy of the smooth muscles, which is most prevalent in the uterus and gastrointestinal tract. However, Leiomyoma originating from smooth muscle at the vessels lying on deep soft tissue is very rare.

**Case Report:**

Our case was a rare case of leiomyoma originating from the axillary region, which was initially diagnosed as a fibroadenoma on radiological examination. The mass was separated from surrounding tissues and totally resected. Pathologically, hematoxylin–eosin-stained biopsy tissue showed the typical findings of leiomyoma. Postoperative follow-up observation was done for 1 year, without any complications or recurrence.

**Conclusion:**

Notably, a leiomyoma in the axillary region is difficult to differentiate from other benign or malignant tumors on preoperative radiological examinations such as ultrasonography or computed tomgraphy. Therefore, when an indefinite asymptomatic mass that is not lymphadenopathy or common benign tumor is identified in the axillary region, leiomyoma can be considered as one of the differential diagnoses.

## 1 Introduction

Leiomyoma is a form of benign tumor originated in hypertrophy of the smooth muscles, which is most prevalent in the uterus and gastrointestinal tract.^[1]^ Leiomyoma, when it develops in the uterus, can accompany with menstrual disorders, infertility, and miscarriage. Its presence in the small intestines can cause digestive disorders and bleeding, with some cases of transformation into malignant tumors, such as sarcoma.^[2]^ Leiomyoma development in deep soft tissues is very rare, with most cases typically originating from the blood vessels.^[3,4]^ Until now, cases involving leiomyoma developing in deep soft tissues have rarely been reported.

Our case involved a leiomyoma that developed in the left axillary region. We hereby report this case because it is rare, as well as an examination of its differential diagnoses through literature review.

## 2 Case report

**Figure 1:**
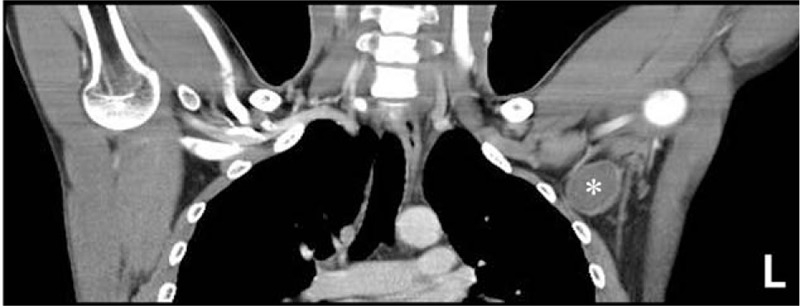
Preoperative CT. A well-demarcated mass of approximately 3.5 × 3.4 cm, with features consistent with those of a fibroadenoma, was observed at low attenuation in the left axillary region, denoted as an asterisk mark (^∗^). CT = computed tomography.

A 29-year-old man noticed a hard, palpable mass of approximately 5 × 5 cm in the left axillary region, which was found by patients 1 week earlier. Although protrusion of the mass was nonvisible, there was no change in the skin texture. The mass showed a pattern of continued growth; however, there were no other complaints of specific discomfort from the patient. It was difficult to determine the characteristics or diagnose the mass through physical examination alone. On preoperative contrast-enhanced computed tomography (CT), a well-demarcated mass of approximately 3.5 × 3.4 cm, with features consistent with those of a fibroadenoma, was observed at low attenuation in the left axillary region (Fig. 1). Patient's informed consent was done preoperatively. Surgical excision was done, and we identified a well-demarcated mass attached to the inner side of the pectoralis major muscle and on the pectoralis minor muscle. The mass was separated from surrounding tissues and totally resected.

**Figure 2:**
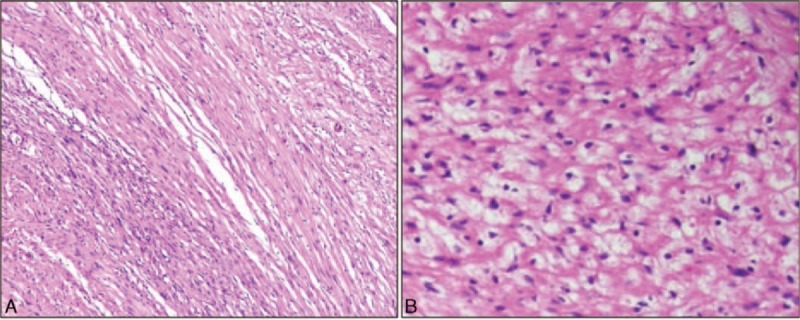
Pathologic findings. A, Irregularly arranged intersecting fascicles of highly differentiated smooth muscle cells are seen (H&E,  ×100). B, Atypia of nuclei and cellular matrix is not seen (H&E,  × 400). H&E = hematoxylin–eosin.

Pathologically, hematoxylin–eosin-stained biopsy tissue showed the arrangement of long intersecting bundles of spindle-shaped cells with relatively uniform oval-shaped nuclei, which corresponded to the typical findings of leiomyoma (Fig. 2A and B). Since the shapes of the nuclei and cells did not show abnormalities, the findings did not suggest malignant transformation. Postoperative follow-up observation was done for 1 year, without any complications or recurrence.

## 3 Discussion

Leiomyoma is a benign tumor that is commonly observed in the uterus, or digestive system.^[1]^ Although the cause of leiomyoma has not been clearly identified, it is suspected to originate from a single smooth muscle cell in the area of occurrence.^[5]^ Leiomyomas have margins that are mostly well demarcated; they appear as round, hard, whitish-gray masses.^[4]^ Generally, no specific symptoms are manifested, although pain may be caused by pressure on the nearby nerves as the mass grows in size.^[6]^

Leiomyomas mostly develop in the abdominal cavity or retroperitoneal cavities such as the uterus; its development in the deep soft tissue is very rare, as are previously documented reports.^[6–9]^ Surgical resection is the treatment of choice, as roughly 0.5% of leiomyomas can undergo malignant transformation to leiomyosarcoma.^[10]^

Leiomyoma in the axillary region is extremely rare, with almost no reported cases.^[11,12]^ Differential diagnosis for imaging diagnosis according to the type of axillary mass is summarized in Table 1. For masses discovered in the axilla, differential diagnoses can include metastatic cancer or lymph node metastasis from breast cancer for malignant cases, and simple lymphadenopathy, inflammation, fibroadenoma, fibroid change, or lipoma for benign cases.^[13]^ For differentiating these masses, the use of imaging tests, such as ultrasonography, CT, and magnetic resonance imaging (MRI), is essential. Among such tests, ultrasonography is the first choice, since it is not only very useful in distinguishing whether the phase of the mass is solid or cystic but also has a low cost, can identify most anatomical structures, and identify the phase of blood flow through the use of a Doppler image.^[14]^

**Table 1:**
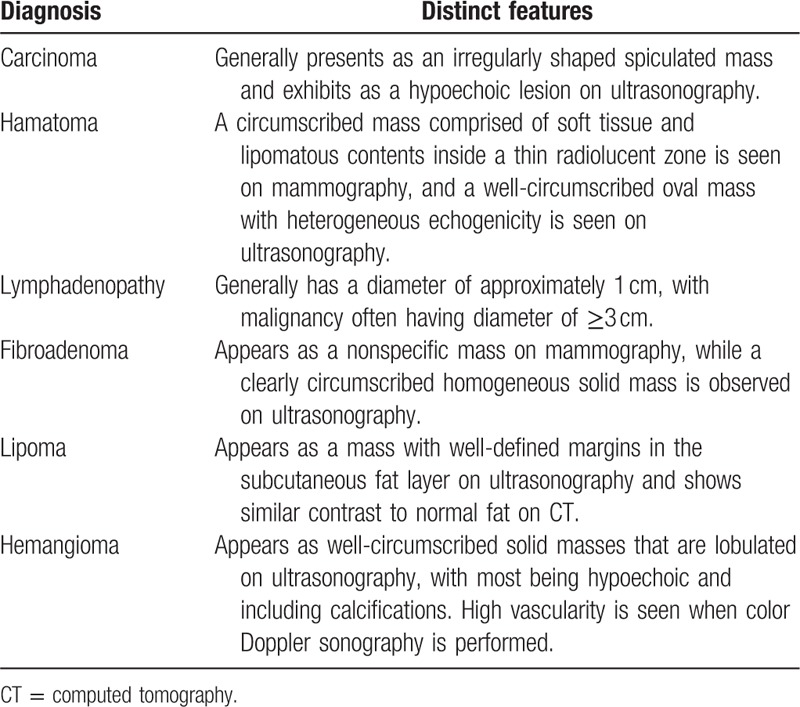
Differential diagnosis of axillary masses.

Although accurate initial diagnosis of axillary leiomyomas is difficult, complete resection is required considering the possibility of malignant lesions and differentiating them from breast cancer or lymph node metastasis. In cases of difficult or ambiguous diagnosis of a deep mass in the axillary region, it can be considered one of the differential diagnoses.
